# A hypertrophic senile heart

**DOI:** 10.1007/s12471-020-01370-9

**Published:** 2020-02-05

**Authors:** I. Arts-Birstonaite, J. Jaspers Focks, C. Camaro, R. Nijveldt

**Affiliations:** Department of Cardiology, Radboud University Medical Center, Nijmegen, The Netherlands

**Fig. 1:**
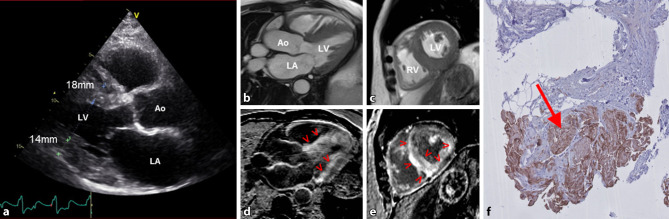
Echocardiographic parasternal long axis view with hypertrophic interventricular septum (18 mm) and posterior wall (14 mm) of the left ventricle (**a**). CMR cine images demonstrated thickened myocardium on the 3‑chamber (**b**) and mid-ventricular short axis (**c**) views, with the corresponding late gadolinium enhanced images (**d**, **e**) showing diffuse subendocardial contrast enhancement *(red arrow heads*). Light microscopy of the septal myocardial biopsy shows Congo red staining positive for amyloid (*arrow*). *CMR* cardiac magnetic resonance, *Ao* aorta, *LV* left ventricle, *LA* left atrium, *RV* right ventricle

In this case report we describe a 77-year-old man with a history of bilateral carpal tunnel syndrome who presented with dyspnoea on exertion. The electrocardiogram showed low voltage QRS complexes, while echocardiography demonstrated prominent concentric hypertrophy of the left ventricle with speckled appearance of the myocardium (Fig. 1a). Coronary angiography revealed severe proximal left anterior descending artery stenosis for which intervention was performed. Since symptoms persisted, cardiac magnetic resonance imaging was performed for further evaluation (Fig. 1b, c). The proper late gadolinium enhancement (LGE) images were difficult to obtain due to the inability to suppress the signal of ‘normal’ myocardium—a pathognomonic phenomenon for diffuse amyloid fibrils deposition in the whole myocardium (Fig. 1d, e). Endomyocardial biopsy verified the diagnosis of transthyretin cardiac amyloidosis (ATTR-CA) with Congo red staining of transthyretin fibrils (Fig. 1f).

ATTR-CA exists in mutated and wild-type (ATTRwt) amyloidosis. This type is underestimated among the elderly, especially in patients with aortic stenosis and secondary left ventricular hypertrophy [1]. Studies show its incidence of 25% in octogenarians [1]. In this case, the concomitant presence of carpal tunnel syndrome could have been a clue.

Besides supportive therapy with diuretics, tafamidis has recently been introduced. This benzoxazole derivate binds to transthyretin fibrils, preventing further dissociation to monomers and depositions in the myocardium. It is particularly effective in the early stage of disease (NYHA class I–II) and in ATTRwt [2].
